# Supplementary material for: Unravelling the room-temperature atomic structure and growth kinetics of lithium metal
Source: Nat Commun. 2020 Oct 23;11:5367. doi: 10.1038/s41467-020-19206-w (PMC7585417; doi:10.1038/s41467-020-19206-w)
Supplement: Supplementary file 1 — Supplementary Information [file 41467_2020_19206_MOESM1_ESM.pdf]

Supplementary Information

Unravelling the room-temperature atomic structure and growth kinetics of lithium metal

Chao Liang[1], Xun Zhang[1], Shuixin Xia[1], Zeyu Wang[1], Jiayi Wu[1], Biao Yuan[1], Xin Luo[1], Weiyan Liu[1], Wei Liu[1] & Yi Yu[1*]

Affiliation:

[1]School of Physical Science and Technology, ShanghaiTech University, Shanghai 201210, China

*Correspondence to: yuyi1@shanghaitech.edu.cn. (Y.Y.)

Movie captions.

Supplementary Movie 1: Beam induced in situ growth of lithium particles from lithium carbonates. (room temperature, 5×speed).

Supplementary Movie 2: Beam induced in situ growth of sodium particles from sodium carbonates. (room temperature, 5×speed).

Supplementary Movie 3: Beam induced in situ growth of lithium particles from lithium fluorides. (room temperature, 2×speed).

Supplementary Movie 4: In situ growth of lithium particles at high spatiotemporal resolution. (room temperature, 0.2×speed).

Supplementary Movie 5: In situ growth of lithium whisker from lithium particle. (5×speed).

Supplementary Movie 6: In situ growth of lithium whisker from lithium fluoride. (5×speed).

Supplementary Movie 7: Cryogenic condition for lithium carbonates under the electron beam. (cryogenic temperature -178 ºC, 5×speed)

Supplementary Movie 8: Cryogenic condition for sodium carbonates under the electron beam. (cryogenic temperature -178 ºC, 5×speed)

Supplementary Movie 9: In situ formation of lithium whisker with the help of an STM tip. (5×speed).

Supplementary Movie 10: Breakdown of lithium whisker. (1×speed).

Supplementary Movie 11: Li-Li contact. (1×speed).

Supplementary Movie 12: Li_2O-Li_2O contact. (1×speed).

Supplementary Movie 13: Li-Li_2O contact. (1×speed).

Analysis of lithium whisker growth rates.

Image stacks in Supplementary Movies 4, 5 were acquired at a frame rate of 5 frames per second. As the exposure time of each image is 0.2 s, the measured lengths can only reflect the changes in this interval. This sampling is enough for the length-time curve, but not that enough for the growth rate-time curve (growth rate is defined as $\Delta length/\Delta t$). Therefore, smoothing is applied to the growth rate-time curve. The raw data without smoothing is shown in Supplementary Fig. 4. The smoothing method is as follows. For each data point, its five nearby points are taken into average with different weights, described as the following equation, $v_t=0.05v_{t-3}+0.15v_{t-2}+0.2v_{t-1}+0.2v_t+0.2v_{t+1}+0.15v_{t+2}+0.05v_{t+3}$. After smoothing, major features can be kept while noise or discontinuity can be removed. To analyze the growth length and growth rate versus time, we adopt the cross coefficients ρ to assess their relationships.

$$\rho = \frac{COV(X,\ Y)}{\sigma_X \sigma_Y} \quad (1)$$

Here $COV(X, Y)$ represents the covariance of random variables X and Y, σ_X and σ_Y represent the corresponding standard deviations. The greater the cross coefficients, the greater the correlation between the two variables. The calculation results are as follows. ρ(TypeI, length-time) = 0.971, ρ(TypeI, rate-time, raw curve) = 0.769, ρ(TypeI, rate-time, smoothed) = 0.993, ρ(TypeII, length-time) = 0.998, ρ(TypeII, rate-time, raw curve) = 0.330, ρ(TypeII, rate-time, smoothed) = 0.854.

Image simulation.

Image simulations (Supplementary Fig. 6) were performed using the multislice method as implemented in MacTempasX[1]. For the simulated aberration-corrected high-resolution transmission electron microscopy (AC-HRTEM) images, the accelerating voltage was 300 kV, C_S value was -10 μm, beam convergence angle was 0.2 mrad, and spread of defocus was 1nm. Other aberrations are not considered (set to zero).

Moiré fringes analysis.

Supplementary Fig. 7 shows the analysis procedure of the Moiré fringes. In principle, the Fourier transformation spots of Moiré fringes originates from the vector difference of different lattice fringes. In our case, this mechanism was revealed in Supplementary Fig. 7b. The vectors of Moiré fringes (yellow arrows) are equal to the vector difference between lithium (red arrows) and lithium oxide (blue arrows). The distribution of lithium and lithium oxide can be seen from their corresponding inverse Fourier transformation images respectively.

Energy filtered TEM (EFTEM).

EFTEM images in Fig. 4 were collected by using three windows method[2]. Electron energy-loss spectroscopy (EELS) collection angles as well as the onsets of lithium and sodium peaks were carefully measured and calibrated before EFTEM imaging. We have collected the EELS of lithium and sodium particles respectively to determine the location of each window. For the EFTEM image of lithium (K-edge), the pre-edge windows were set as 41-47 eV and 47-53 eV, the post-edge window was set as 55-61 eV. For the EFTEM image of sodium (L-edge), the pre-edge windows were set as 23-27 eV and 27-31 eV, the post-edge window was set as 32-36eV. EFTEM image of oxygen could not be obtained as oxygen peak ~530 eV could not be distinguished, and long-time exposure for the high-energy core-loss signal would damage the particles.

Thickness measurement.

We computed the thickness *t* of the lithium and sodium particles by equation as following[2]:

$$\frac{t}{\lambda} = \ln\left(\frac{I_t}{I_0}\right) \quad (2)$$

where I_t represents the integral area of the total EELS spectrum, I_0 represents the integral area of the zero-loss peak, λ is the inelastic mean free path (IMFP) of the electrons passing through the lithium and sodium particles. EFTEM relative thickness mapping was achieved by dividing a TEM image by a zero-loss image and taking the logarithm of it[3]. Absolute thickness map was obtained by taking the calculated IMFP into account. The results are shown in Supplementary Fig. 9. We measured the average thickness in yellow boxes and the line thickness profiles can be seen, respectively. Typically, the thickness of lithium particles is 30-70 nm and the average thickness is ~60 nm. The thickness of sodium particles is 10-40 nm and the average thickness is ~30 nm.

Oxide layer formation.

To investigate the formation condition of the oxide layer, we design beam blanked experiments to exclude the influence of electron beam. The results are shown in Supplementary Fig. 10, local details are magnified in two corresponding squares. Several alkali metal particles grew up previously, and then the beam was blanked during the formation of the oxide layer. These particles were exposed 1s every five minutes to minimize the influence of electron beam as much as possible. Our results show that though under this condition, the formation of oxide layer can still be seen which indicates the oxidation behavior is not induced by the electron beam. The thickness of oxide layers increased as time went on. In the same manner, Supplementary Fig. 5 shows the situation of lithium particles grown out from lithium fluoride. As can be seen clearly, the formation of lithium particles is earlier than the formation of lithium oxide. Surprisingly, although there is no oxygen element in this system, the oxidation behavior can still be observed after the formation of lithium particles.

Oxide distribution and the origin of oxidation.

We utilized both energy-dispersive X-ray spectroscopy (EDS) and EELS to investigate the distribution of oxide. TEM-EDS results are shown in Supplementary Fig. 11. We collected the EDS signals of the initial state and oxidized state of the sample with the same acquisition time (~5 min). The circles represent the area exposed to the electron beam. The area was chosen that the supporting membrane did not appear in the field of view so that the influence of carbon element can be excluded. As can be seen from the spectra, five minutes after the formation of lithium particles, the counts of oxygen element increased obviously. This indicates the strong adsorption of environmental trace oxygen by alkali metals.

Scanning transmission electron microscopy (STEM)-EDS and STEM-EELS experiments were carried out under the cryogenic condition as alkali metal particles were easily damaged by the STEM probe, so that sample cooling was applied to slow down the structure collapse. To prevent the heavily carbon contamination in STEM mode, the sample had been beam-showered firstly. Then the formation of lithium particles was performed under the TEM mode as mentioned in the main text. Later, the sample was cooled down to -178 ºC and the status was maintained for 40 minutes to wait for thermal equilibrium and to avoid sample drift. Supplementary Fig. 12 shows the STEM-EDS results. After 40 minutes, lithium particles formed on the surface of the LiF particle were oxidized. (Because alkali particles can be oxidized easily in several minutes before they had been cooled down.) No obvious carbon contamination can be observed. As a reference, no obvious signal of oxygen element can be seen from the supporting carbon membrane. STEM-EDS mapping agrees with previous TEM-EDS results, and it can be further confirmed by the STEM-EELS results (Supplementary Fig. 13). Obvious oxygen signals can be detected on the edge of the lithium particles.

Furthermore, to confirm whether the oxygen element comes from the oxygen adsorption of

supporting carbon membrane or not, experiments on bare gold and nickel TEM grids without supporting membranes were compared. The results are shown in Supplementary Fig. 14 and the appearance of lithium oxide can still be identified. Hence, we deduce that the trace oxygen may come from the TEM column even it is under the high vacuum condition.

In situ heating experiments.

Heating experiments were carried out to explore the mechanism of alkali metal formation. Considering that the melting point of bulk lithium metal is 180 ºC under atmospheric pressure and could be lower in high vacuum, the testing temperature range for heating experiments was set from 40 ºC to 200 ºC. The results are shown in Supplementary Fig. 15. We heated the samples gradually at a rate of 10 ºC per minute. To minimize the impact of the electron beam, observations were carried out in the way that samples were exposed to the electron beam for 1 s every 1 min. Even though the sample had been finally heated to 200 ºC, generation of lithium particles could not be observed all the way. For comparison, the samples were cooled back to 40 ºC and irradiated to the electron beam for dozens of seconds, then the formation of lithium particles could be observed. Therefore, the heating effect may not be the main driving force of the formation of alkali metal particles.

First principle calculations.

The study of the thermodynamic decomposition of Li_2CO_3, Na_2CO_3 and LiF was carried out by using the first-principles calculation based on the density functional theory (DFT), which was implemented in the Vienna ab initio simulation package (VASP)[4] with the projector-augmented wave (PAW) potentials[5]. The generalized gradient approximation (GGA) of Perdew-Burke-Ernzerhof (PBE) was chosen for the exchange-correlation energy[6]. An energy cutoff of 500 eV

was set to ensure a good convergence, and the Brillouin zone was sampled by the Monkhorst-Pack scheme for metals and tetrahedron method with Blöchl corrections for others. A 4×6×6 k-point grid was used for Li_2CO_3 and Na_2CO_3 models, containing 8 metal (Li or Na) atoms, 4 carbon atoms and 12 oxygen atoms. A 6×6×6 k-point grid was used for LiF (NaCl-type structure), containing 4 Li and 4 F atoms. A 6×6×6 k-point grid was used for Li_2O and Na_2O models, containing 8 metal (Li or Na) atoms and 4 oxygen atoms. An 8×8×8 k-point grid was used for Li and Na models, containing 2 metal (Li or Na) atoms. The geometry optimization was performed using a conjugate gradient scheme with an energy convergence criterion of 10^{-5} eV and a force convergence criterion of 0.01 eV $Å^{-1}$.

The contact property of lithium metal and surface oxide layer was also studied by DFT. The interfacial work of adhesion W_{ad} was calculated to investigated the interaction between two surfaces. The interfacial work of adhesion is defined as $W_{ad} = (E_{interface} - E_{surface\text{-}1} - E_{surface\text{-}2}) / 2S$, where $E_{interface}$ is the energy of the interfacial supercell which consists of two surfaces labeled as surface-1 and surface-2, and $E_{surface\text{-}1}$ and $E_{surface\text{-}2}$ are the energy of surface-1 and surface-2 respectively. S is the area of the interface, and the factor 2 divided in the equation is due to the two interfaces in one interfacial supercell. The relaxed interface structures including Li(110)-Li(110), Li_2O(001)-Li_2O(001) and Li(001)-Li_2O(001) were displayed in Figs. 6k-n. The interfacial work of adhesion was calculated to be -1.01 J m^{-2} for Li(110)-Li(110), -7.92 J m^{-2} for Li_2O(001) -Li_2O(001), -1.21 J m^{-2} for Li(001)-Li_2O(001) with Li-Li contact at the interface, and -6.43 J m^{-2} for Li(001)-Li_2O(001) with Li-O contact at the interface. The negative interfacial work of adhesion suggests that the two surfaces would spontaneously recombine into an interface if they are close enough.

For the evaluation of thermodynamic decomposition of Li_2CO_3, Na_2CO_3 and LiF, the Gibbs free energy of solids[7] is approximated by the DFT total energy of solid bulk at 0 K, which is

described as:

$$G_s(T) \approx E_s^{DFT} \tag{3}$$

The Gibbs free energy of gas at atmospheric pressure[7] is calculated by the following formula:

$$G_g(T) = E_g^{DFT} + \Delta H(T) - TS_m(T) \tag{4}$$

where E_g^{DFT} is the DFT total energy of an isolated gas molecule at 0 K, $\Delta H(T)$ is the enthalpy difference for the gas molecule that is related to a temperature change between 0 K and T, and $S_m(T)$ is the standard entropy of gas at temperature T. For ideal gas, the enthalpy difference[7] is approximated by:

$$\Delta H(T) \approx nk_{\mathrm{B}}T \tag{5}$$

where n is 7/2 for both CO_2 and O_2, and k_{B} is Boltzmann constant. The equation of calculated standard entropy is that:

$$S_m(T) = S_m(298.15\ \mathrm{K}) + \int_{298.15\ \mathrm{K}}^{T} \frac{C_{p,m}}{T} dT \tag{6}$$

where S_m(298.15K) is the standard entropy of gas at 298.15K, and $C_{p,m}$ is heat capacity of gas that can be estimated by a semi-empirical formula $C_{p,m} = a+bT+cT^2$. For CO_2, S_m(298.15K) = 0.21374 kJ·mol^{-1}·K^{-1} and $C_{p,m}$ = (26.75+0.042258T-0.000014257T^2)×10^3 kJ mol^{-1} K^{-1} at the temperature between 300 and 1500K[8]. For O_2, S_m(298.15K) = 0.205138 kJ mol^{-1} K^{-1} and $C_{p,m}$ = (28.17+0.006297T-0.000007494T^2)×10^{-3} kJ mol^{-1} K^{-1} at the temperature between 273 and 3800 K[8]. For F_2, S_m(298.15K) = 0.20278 kJ mol^{-1} K^{-1} and $C_{p,m}$ = (25.16083+0.02513T−0.0000141244T^2) × 10^3 kJ mol^{-1} K^{-1} at the temperature between 200 and 700 K[9]. In the condition of same temperature with lower pressure (about 10^{-8} kPa in TEM), the Gibbs free energy of gas is calculated by:

$$\Delta G_p = \Delta G_{p_m} + nRT ln \frac{P}{p_m} \tag{7}$$

where p and p_m are the pressure in TEM environment and atmospheric pressure, respectively. n is the amount of substance and R is molar gas constant (8.3145×10^{-3} kJ mol^{-1} K^{-1}).

The plausible reaction paths of Li_2CO_3 decomposition are:

$$Li_2CO_3(s) \rightarrow Li_2O(s) + CO_2(g),$$

$$Li_2O(s) \rightarrow 2Li(s) + \frac{1}{2}O_2(g),$$

$$\text{or } Li_2CO_3(s) \rightarrow 2Li(s) + \frac{1}{2}O_2(g) + CO_2(g),$$

$$2Li(s) + \frac{1}{2}O_2(g) \rightarrow Li_2O(s),$$

the possible reaction path of Na_2CO_3 decomposition is:

$$Na_2CO_3(s) \rightarrow Na_2O(s) + CO_2(g),$$

$$Na_2O(s) \rightarrow 2Na(s) + \frac{1}{2}O_2(g),$$

and the potential reaction path of LiF decomposition is:

$$LiF(s) \rightarrow Li(s) + \frac{1}{2}F_2(g).$$

Hence, the thermodynamic driving force for the decomposition of Li_2CO_3, Na_2CO_3 and LiF, such as the Gibbs free energy of reaction (ΔG), could be calculated by the aforementioned calculations, which is shown in Supplementary Fig. 16. The calculated Gibbs free energy of reaction $Li_2CO_3(s) \rightarrow Li_2O(s) + CO_2(g)$ at 298.15 K is 147.12 kJ mol^{-1}, which is consistent with previous reports[7]. Taking the decomposition of Li_2CO_3 as an example, it can be seen that the threshold temperature for Li_2CO_3 thermal decomposition into Li_2O and CO_2 is more than 800 K at atmospheric pressure (black dashed line) and is more than 500 K at the pressure of 10^{-8} kPa (black full line). Moreover, the threshold temperature for Li_2O thermal decomposition into Li and O_2 is even much higher. However, formation of Li crystals was observed at room temperature in our experiments. Therefore, thermal decomposition could not explain this phenomenon, suggesting that the high energy electron-sample interaction could be the dominant

reason. The same principle can be applied to other reactions. To be noted that, the calculations underestimate the change of Gibbs free energy during a reaction. The thermal decomposition threshold temperatures for Li_2CO_3 and Na_2CO_3 at atmospheric pressure were measured to be higher than 1000 K in experiments[10]. The underestimation can explain the negative value of Gibbs free energy change for the reaction $Na_2O(s) \rightarrow 2Na(s) + \frac{1}{2}O_2(g)$. In addition, it further confirms that thermal decomposition should not be the predominant reaction mechanism.

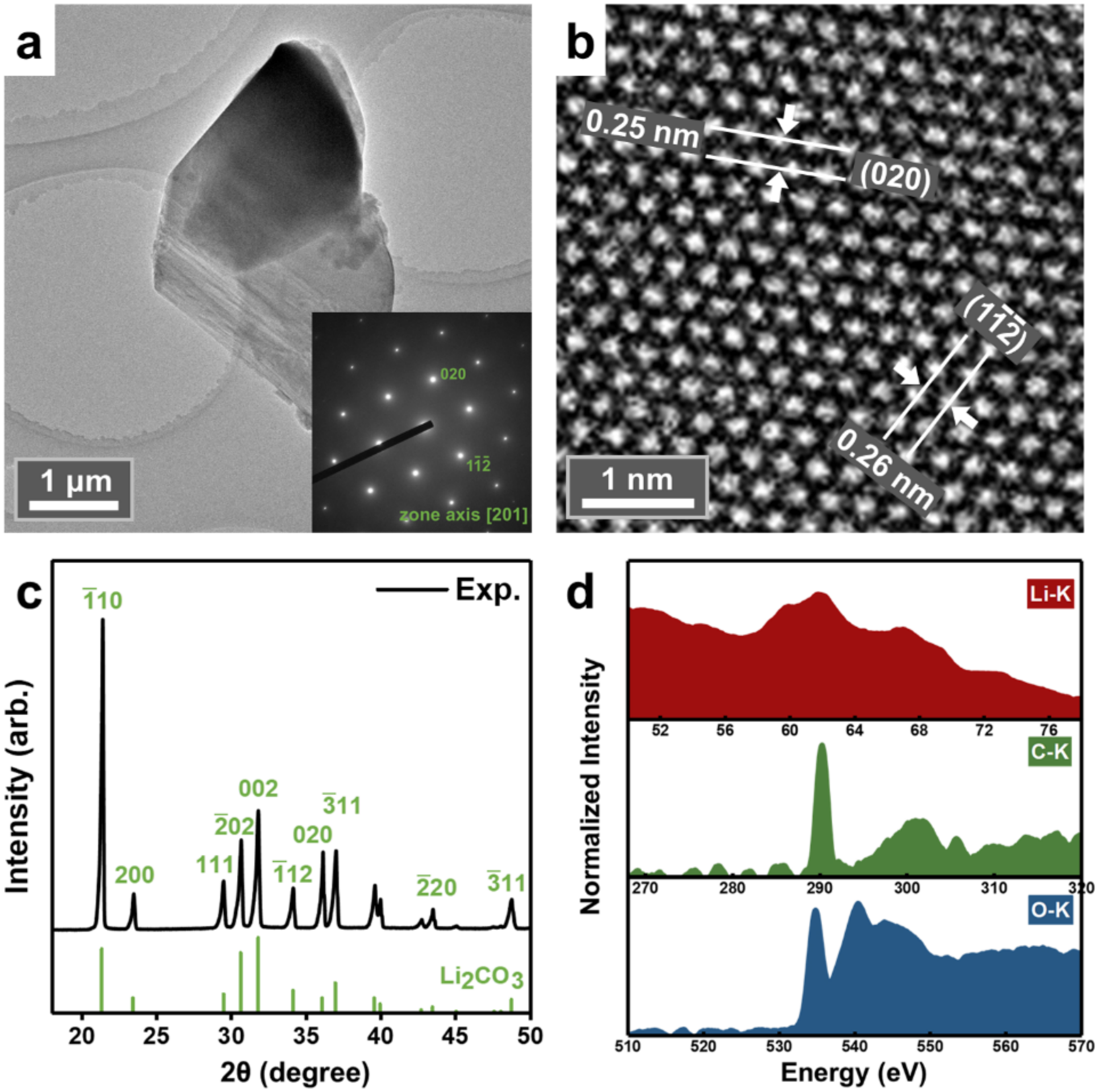

Supplementary Fig. 1 Characterization of Li_2CO_3. a, Morphology of a Li_2CO_3 particle and its SAED as inset. **b**, HRTEM of the particle shows perfect match with its SAED. **c**, Powder x-ray diffraction patterns of Li_2CO_3. **d**, EELS of Li (K-edge), C (K-edge), and O (K-edge) from Li_2CO_3.

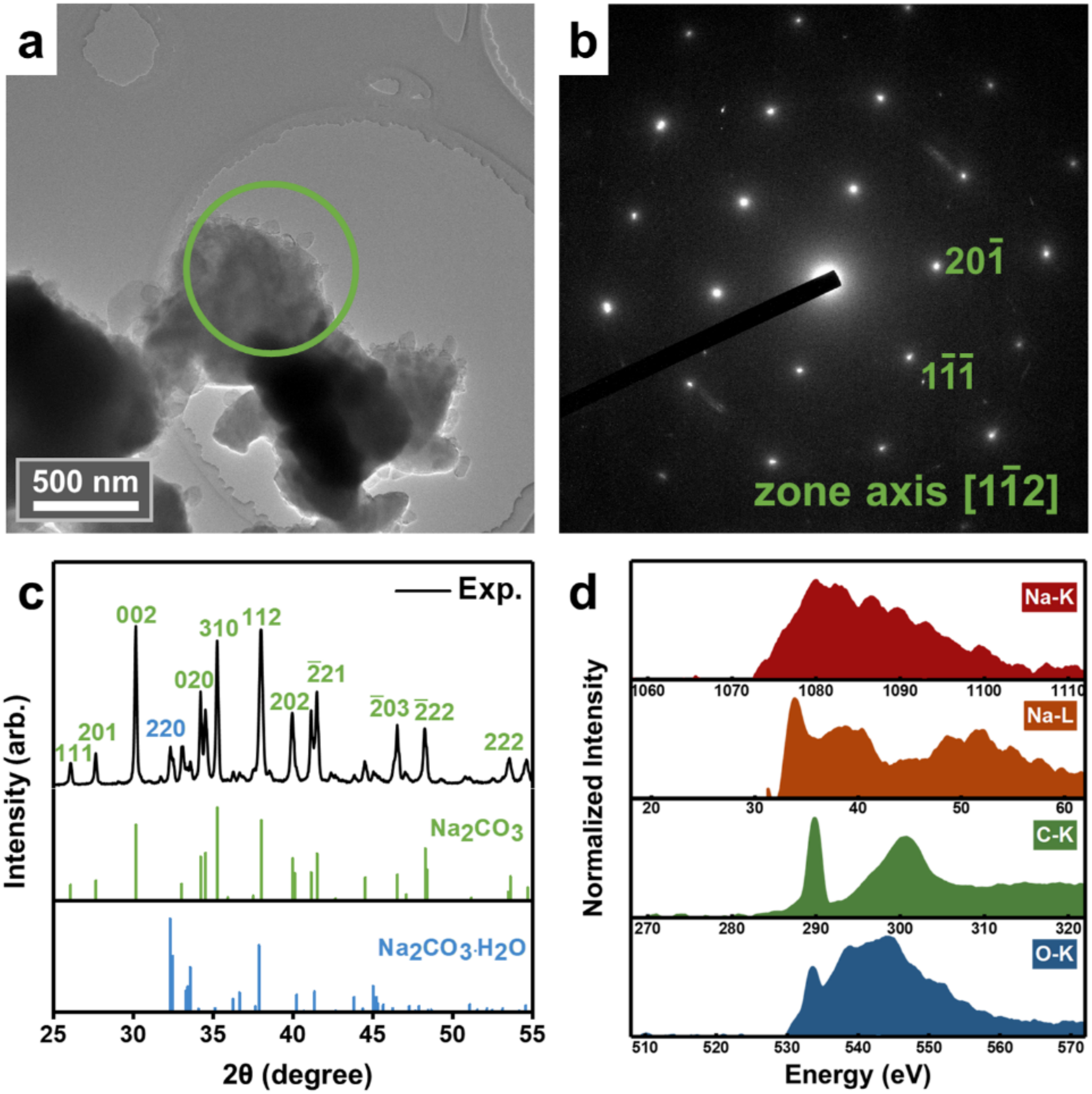

Supplementary Fig. 2 Characterization of Na_2CO_3. a, Morphology of a Na_2CO_3 particle (green circle represents selection aperture) and **b**, its SAED. **c**, Powder x-ray diffraction patterns of Na_2CO_3. **d**, EELS of Na (K-edge, L-edge), C (K-edge), and O (K-edge) from Na_2CO_3.

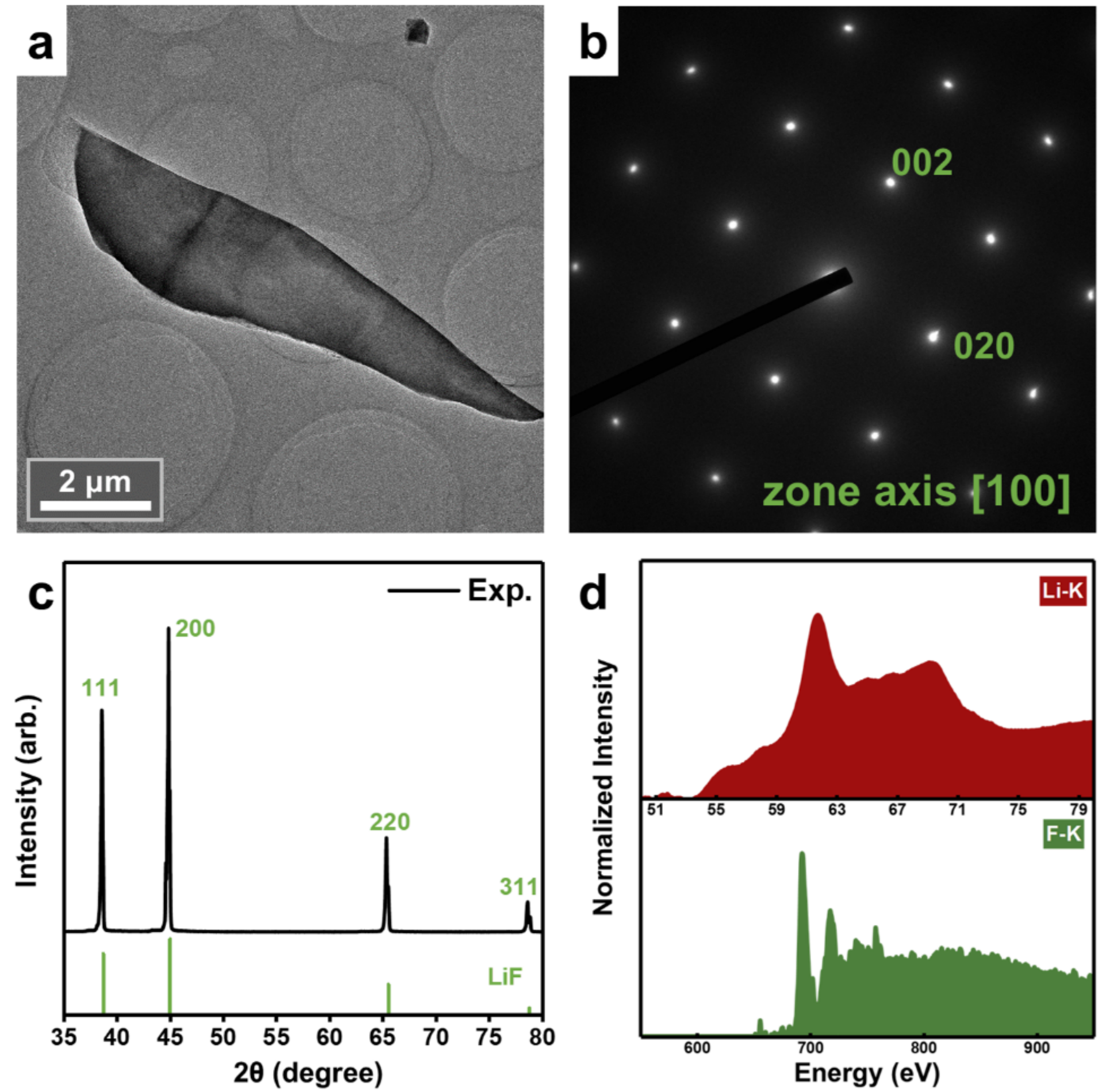

Supplementary Fig. 3 Characterization of LiF. a, Morphology of a LiF particle and **b**, its SAED. **c**, Powder x-ray diffraction patterns of LiF. **d**, EELS of Li (K-edge), F (K-edge) from LiF.

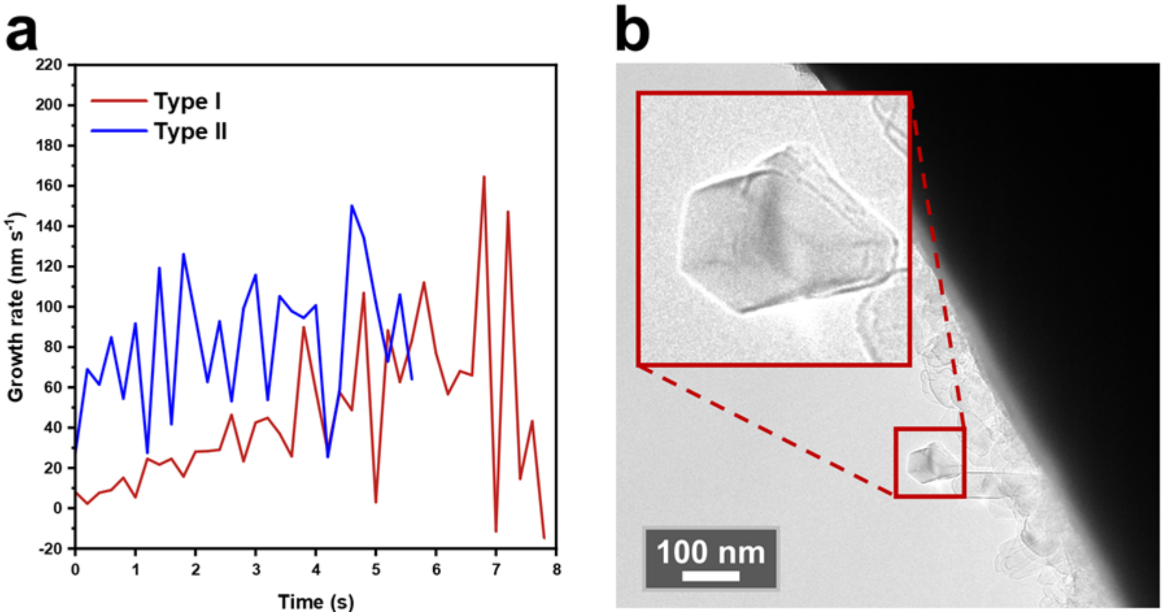

Supplementary Fig. 4 The Growth rate versus time without smoothing in (a) and an image of the cross-section of a lithium whisker in (b).

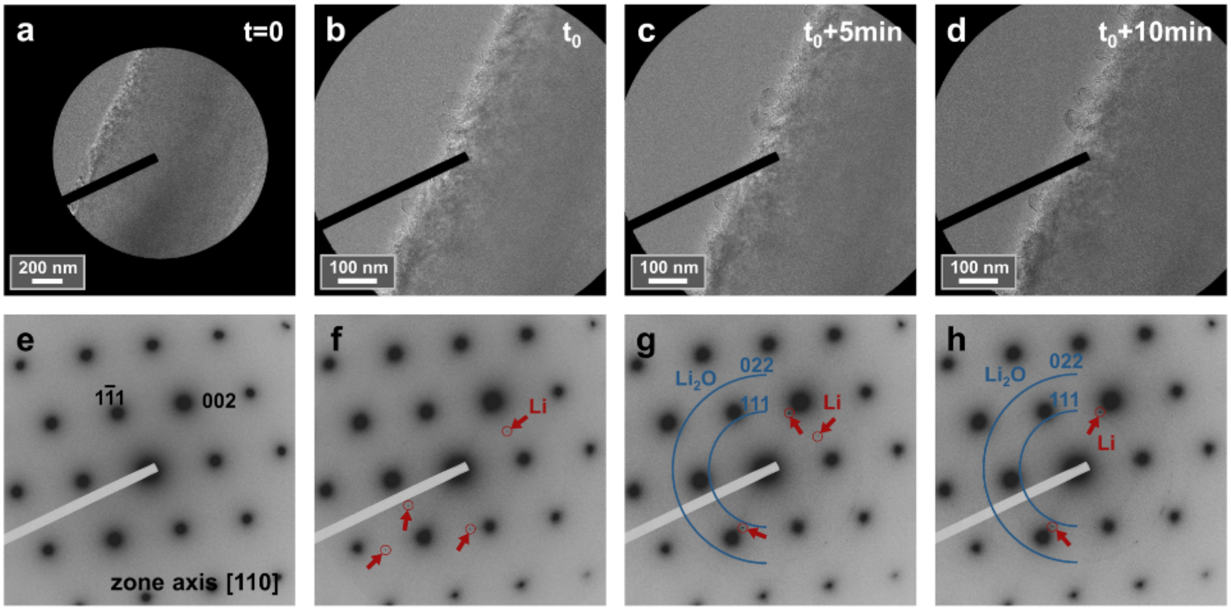

Supplementary Fig. 5 Oxidation process of lithium particles grown out from LiF. a-d, Morphology evolution of the selected area where the black circles represent the selected area apertures. **e-h**, Corresponding SAED patterns. Only the pattern of LiF along [110] axis existed at the beginning (e). After the appearance of patterns of Li (f), the polycrystalline rings of Li_2O appeared later on (g, h), which proves that Li_2O was formed owing to the oxidation of Li.

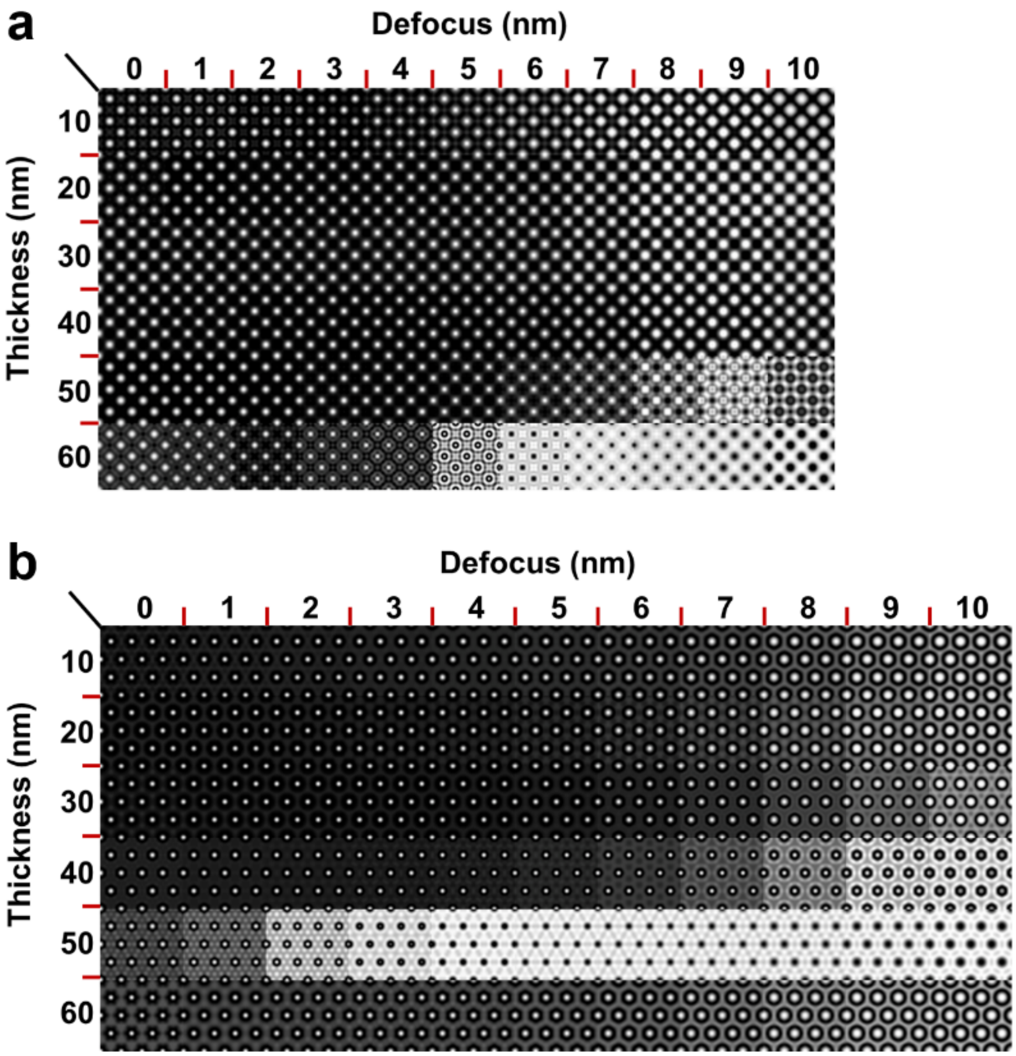

Supplementary Fig. 6 Simulated AC-HRTEM images with different defocus and thickness values. a, Simulated images of lithium. **b**, Simulated images of sodium.

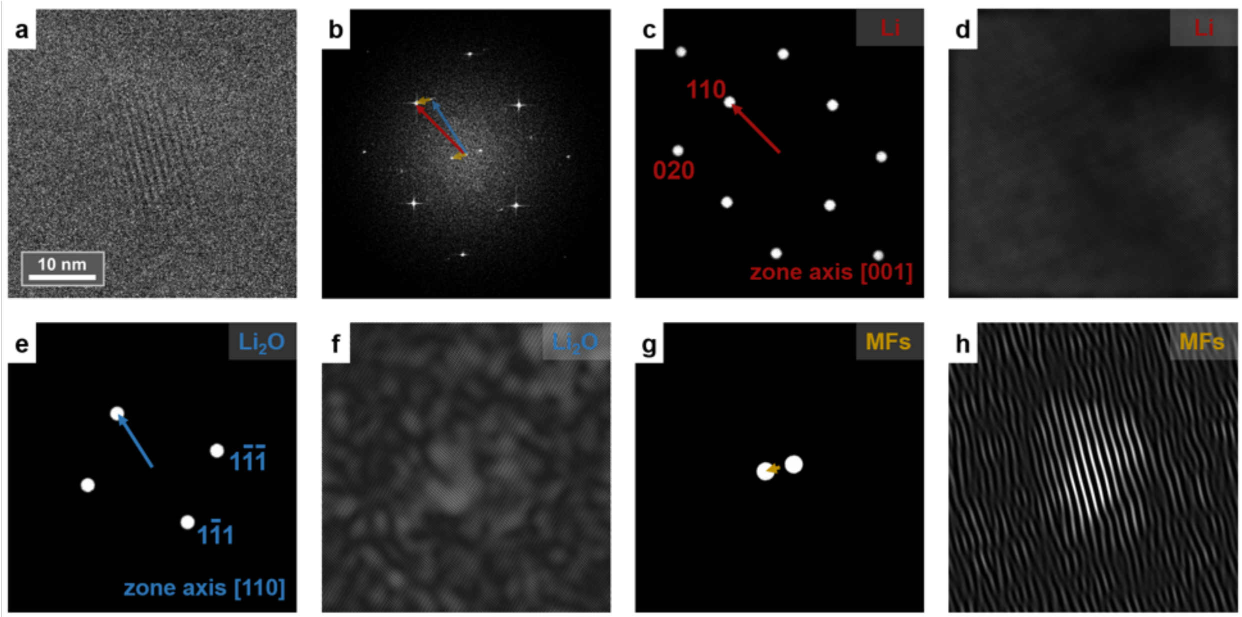

Supplementary Fig. 7 Moiré fringes analysis. **a**, Magnified image of the area with Moiré fringes and its Fourier transformation image in **b**. **c**, Selected spots of lithium composition and **d**, its corresponding inverse Fourier transformation image. **e**, Selected spots of lithium oxide composition and **f**, its corresponding inverse Fourier transformation image. **g**, Selected spots produced by Moiré fringes and **h**, its corresponding inverse Fourier transformation image.

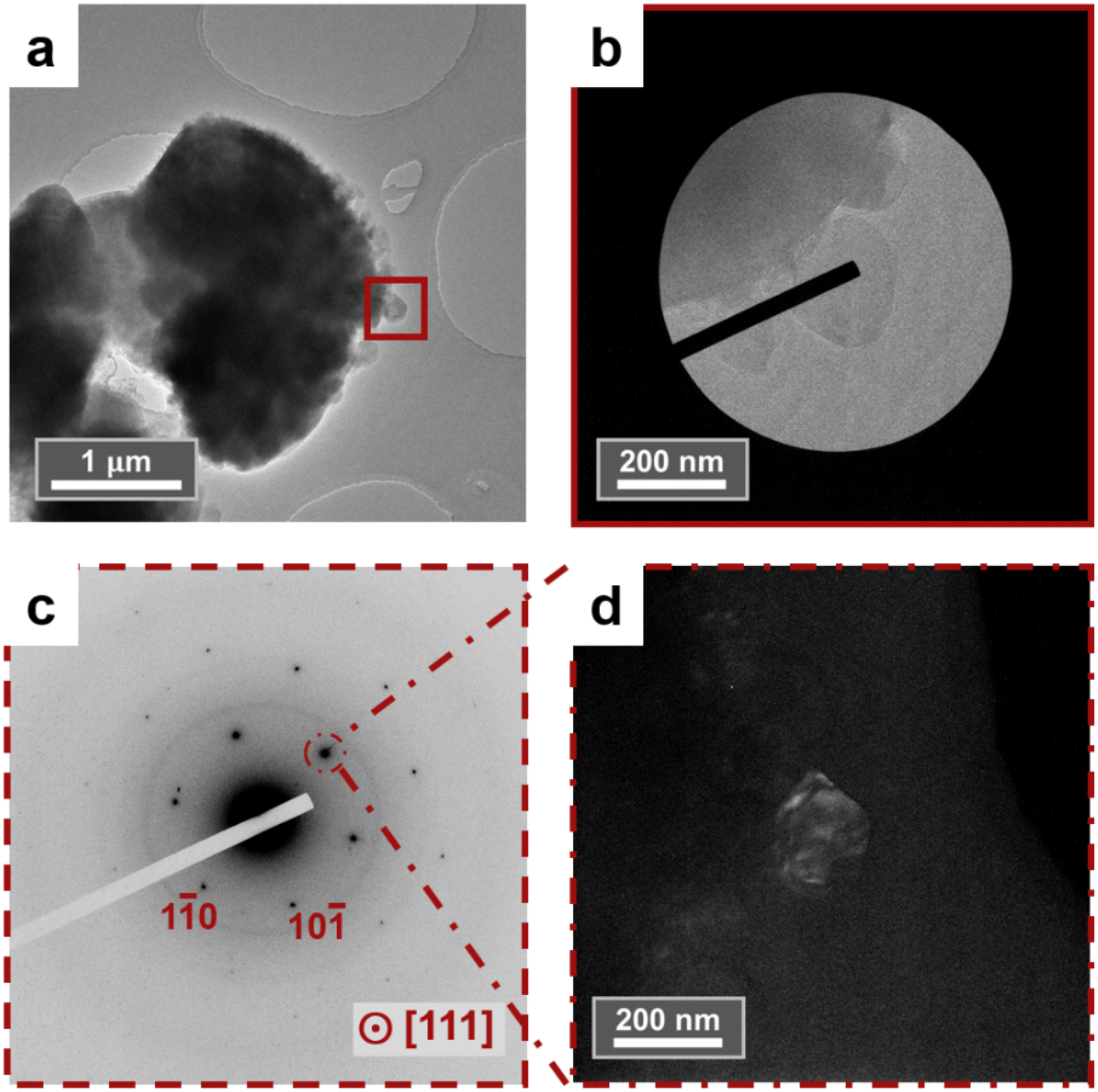

Supplementary Fig. 8 SAED of a sodium particle. **a**, Morphology of sodium carbonate particles. Several sodium particles can be observed at the surfaces. **b**, Selected area of a sodium particle and its original position was marked in **a** with red square. **c**, SAED of this area and corresponding dark field image in **d**.

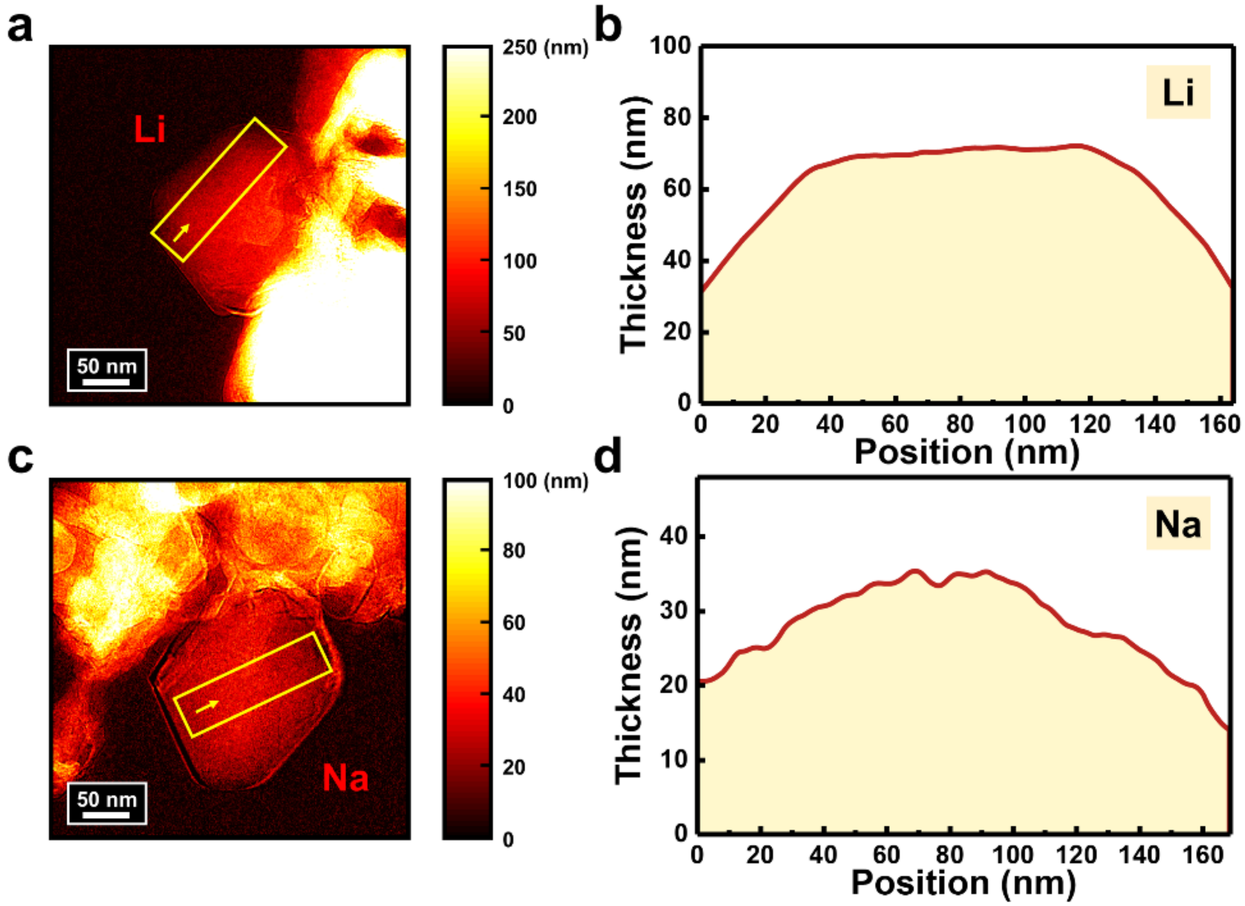

Supplementary Fig. 9 Thickness of the particles. **a**, Thickness map of a lithium particle grown out from lithium carbonate. **b**, Thickness line profile measured along the yellow box in **a**. **c**, Thickness map of a sodium particle grown out from sodium carbonate. **d**, Thickness line profile measured along the yellow box in **c**.

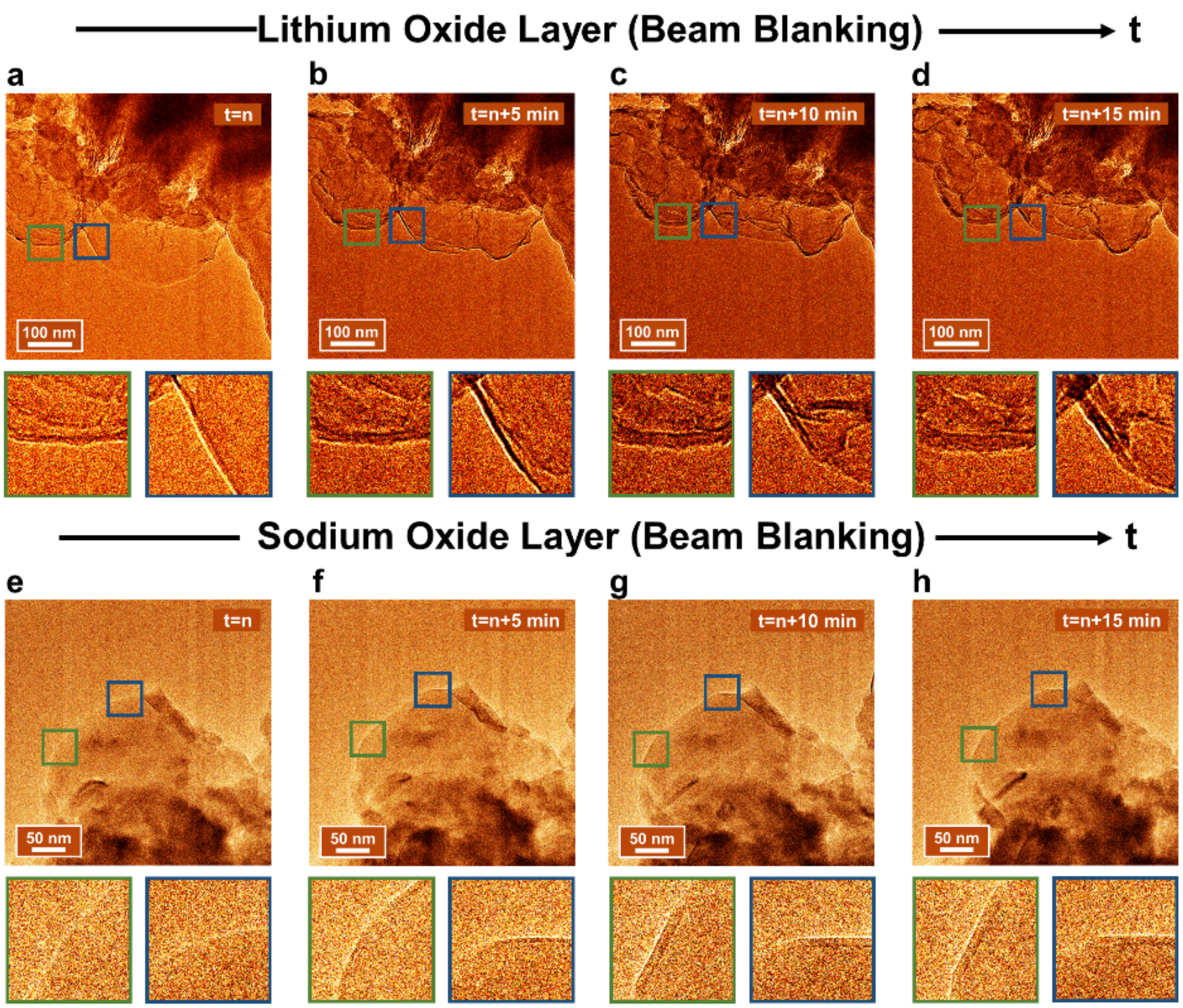

Supplementary Fig. 10 Oxidation process of alkali metal particles. a-d, Snapshots of oxidation process of lithium particles. Beam is blanked during each two images. Two regions in each image are enlarged and showed in corresponding squares. **e-h**, Snapshots of oxidation process of sodium particles. Beam is blanked during each two images. Two regions in each image are enlarged and showed in corresponding squares.

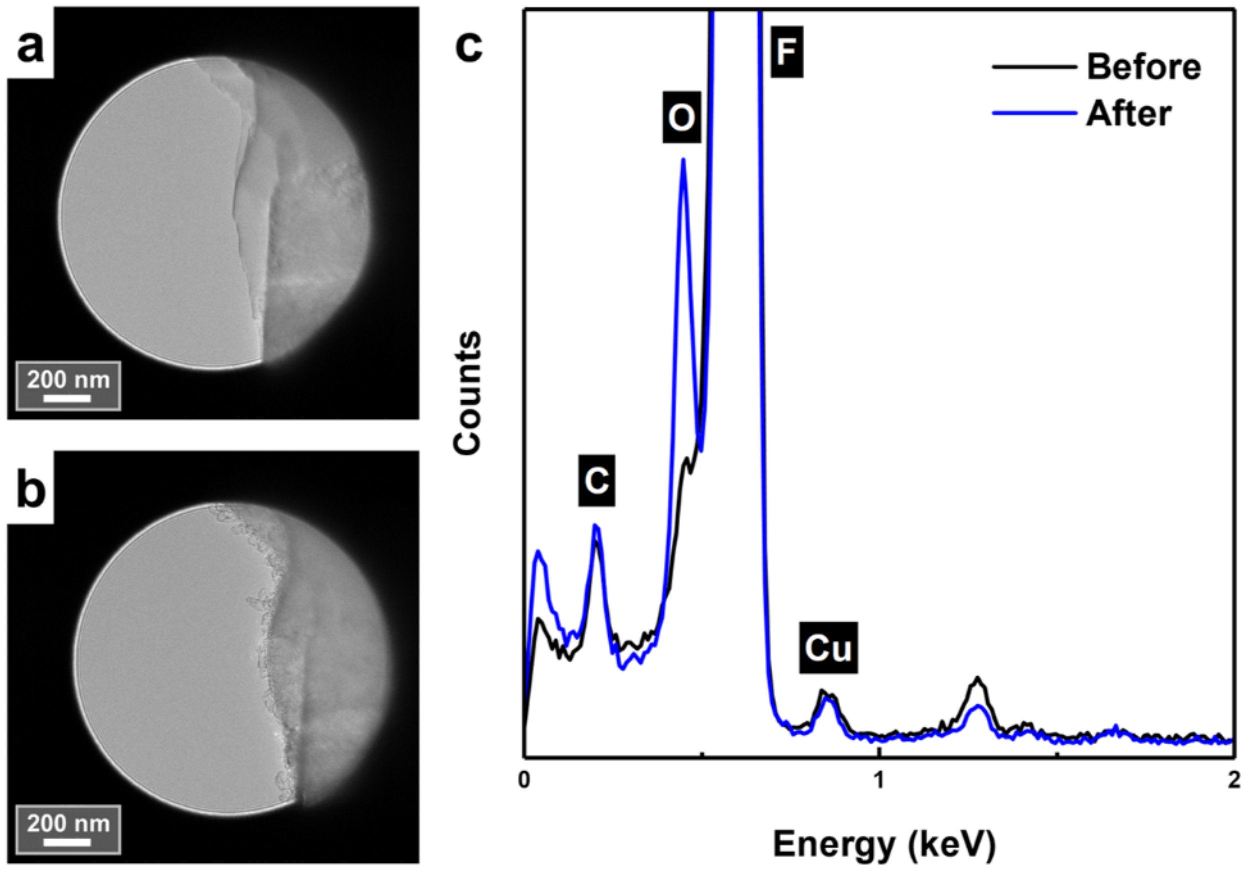

Supplementary Fig. 11 TEM-EDS experiments before and after oxidation. **a**, An area chosen from a LiF particle and its initial morphology. The black circle represents the area irradiated by the electron beam. **b**, The same area after the growth and oxidation of lithium. **c**, Corresponding TEM-EDS spectra collected from this area at initial state and after oxidation.

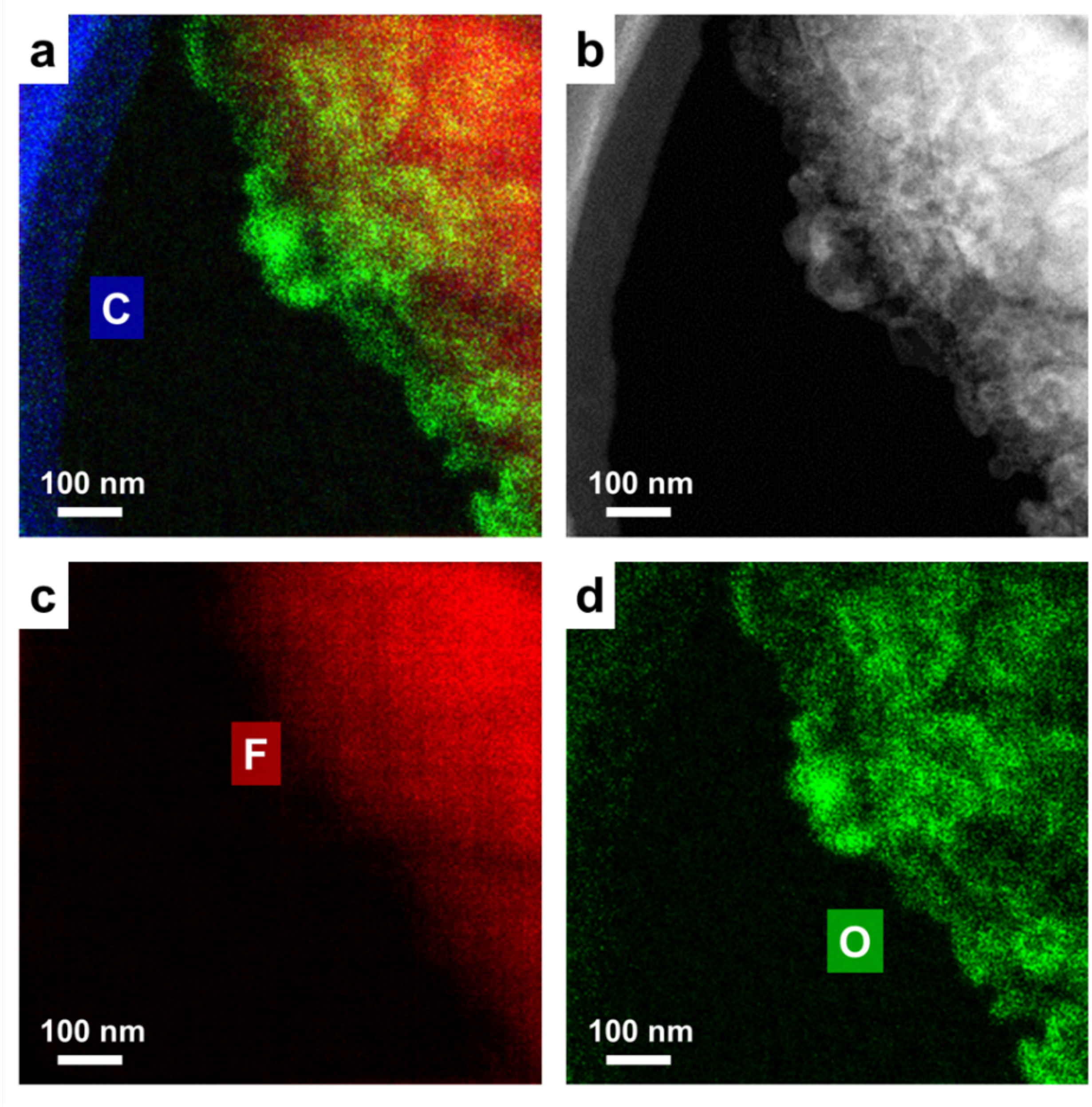

Supplementary Fig. 12 STEM-EDS map of LiF particle with Li particles after oxidization. **a**, Overlapped elemental distribution map with C (blue), F (red) and O (green). **b**, STEM image. **c**, Elemental distribution map of F. **d**, Elemental distribution map of O.

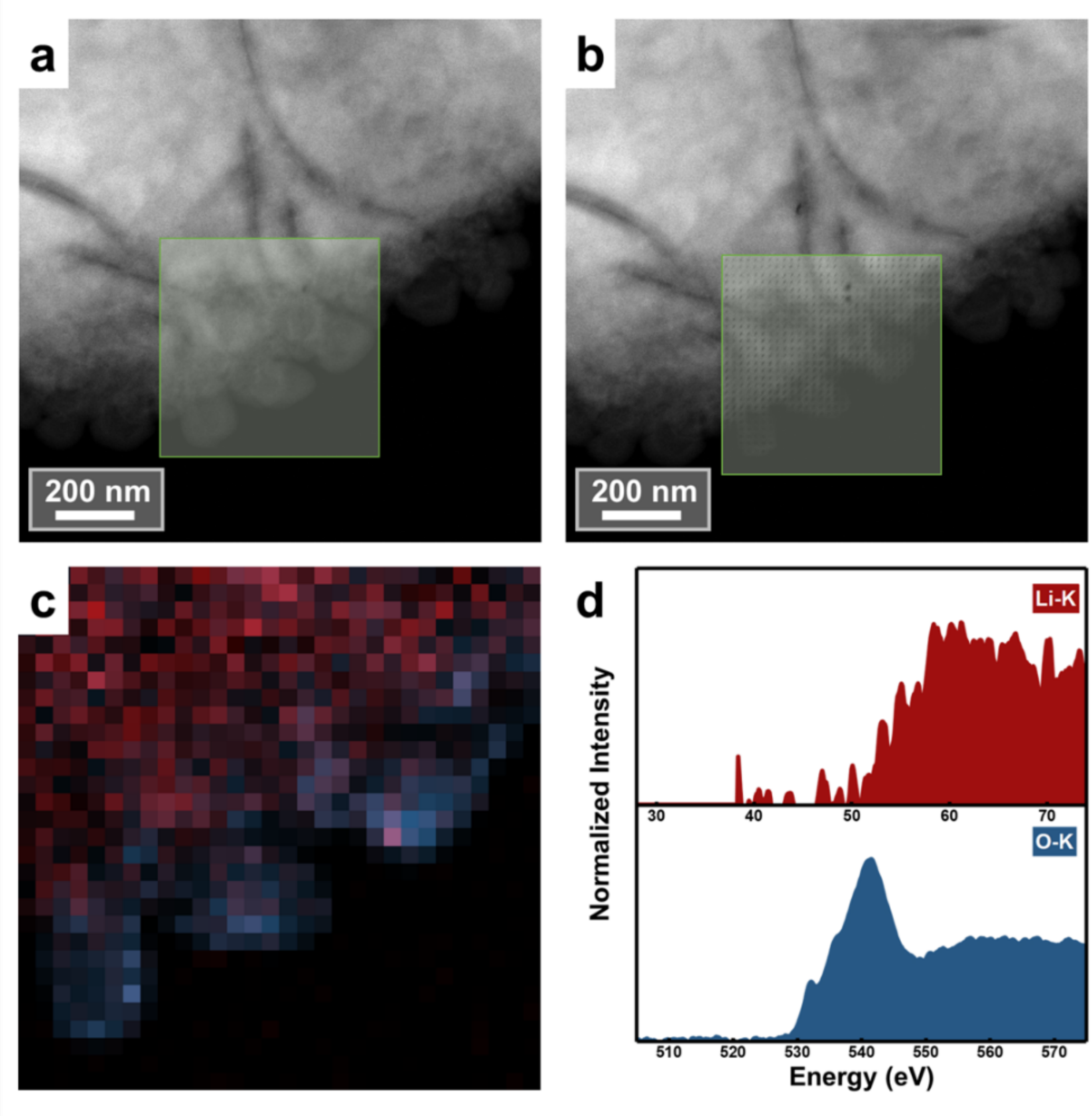

Supplementary Fig. 13 STEM-EELS map of LiF particle with Li particles after oxidization. **a**, Morphology of the LiF particle and surface Li particles. Green square shows the initial morphology of the area at which the EELS has been collected. **b**, Morphology of this particle and Li particles after EELS collection. Periodic spots inside the green square represent the scanning probe positions. **c**, STEM-EELS map of Li (red) and O (blue). **d**, Corresponding spectra of Li and O elements.

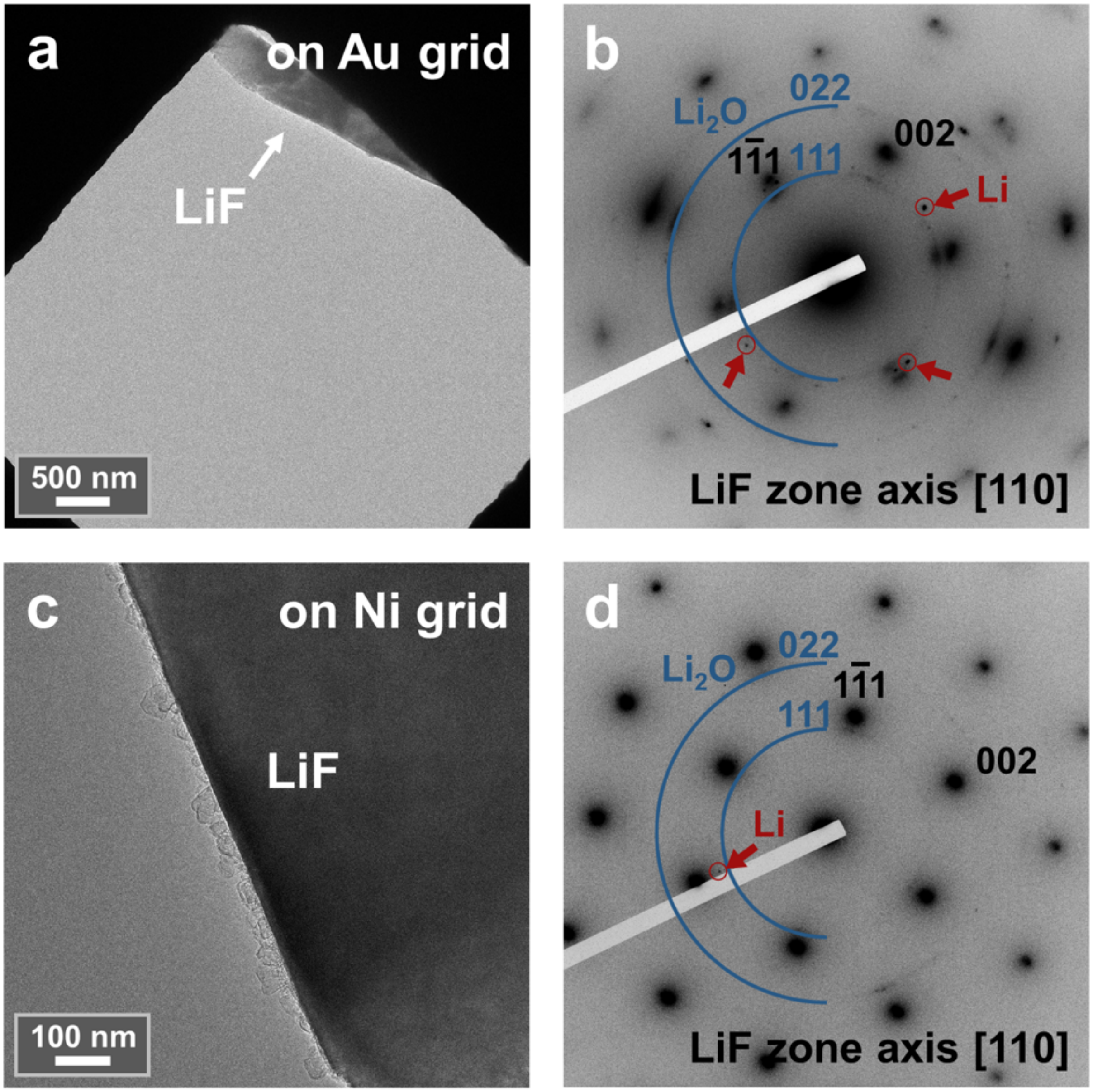

Supplementary Fig. 14 Comparison experiments on bare Au and Ni TEM grids. a, a LiF particle along [110] axis supported on the Au grid without carbon membrane. **b**, SAED patterns of the final products with the presence of Li_2O. **c**, a LiF particle along [110] axis supported on the Ni grid without carbon membrane. **d**, SAED patterns of final products with the presence of Li_2O.

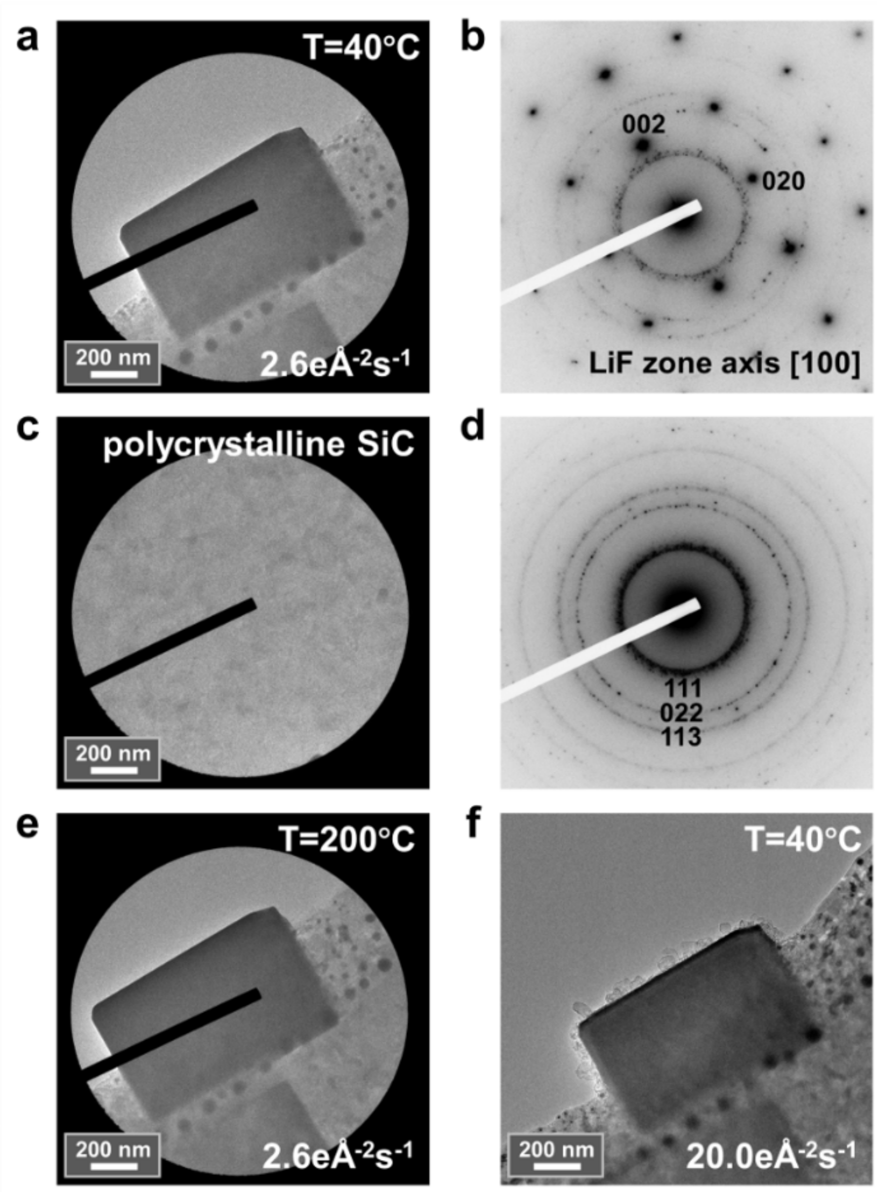

Supplementary Fig. 15 In situ heating experiment. a, A square shape LiF particle along [100] axis at 40 ºC, supported on the polycrystalline SiC membrane and its SAED pattern in **b**. **c**, Pure SiC membrane and its SAED pattern in **d** to demonstrate that the polycrystalline rings come from the SiC substrate. **e**, The particle was heated to 200 ºC and no Li particles could be observed all the way. All these images and diffractions were taken at low dose-rate with short acquisition time. **f**, After the temperature cooled back to 40 ºC, the particle was irradiated at a higher dose-rate. Formation of Li particles can be observed after several dozens of seconds.

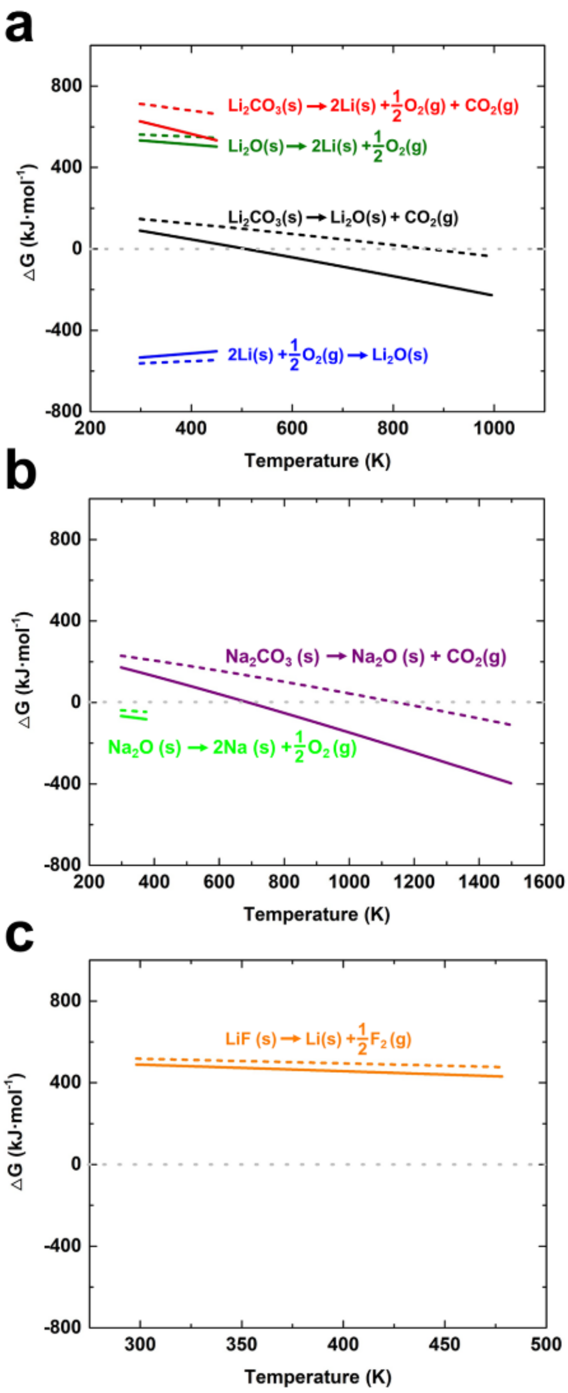

Supplementary Fig. 16 The Gibbs free energy of plausible thermal decomposition reaction paths of a, Li_2CO_3, b, Na_2CO_3 and c, LiF. The dotted lines represent conditions under atmospheric pressure and the solid lines represent conditions in high vacuum.

References:

1 Kilaas, R. MacTempasX. http://www.totalresolution.com (Accessed, 2015).

2 Egerton, R. F. *Electron energy-loss spectroscopy in the electron microscope*. (Springer Science & Business Media, 2011).

3 Hofer, F., Grogger, W., Warbichler, P. & Papst, I. Quantitative energy-filtering transmission electron microscopy (EFTEM). *Microchim. Acta* **132**, 273-288 (2000).

4 Kresse, G. & Furthmüller, J. Efficient iterative schemes for ab initio total-energy calculations using a plane-wave basis set. *Phys. Rev. B* **54**, 11169-11186 (1996).

5 Blöchl, P. E., Jepsen, O. & Andersen, O. K. Improved tetrahedron method for Brillouin-zone integrations. *Phys. Rev. B* **49**, 16223-16233 (1994).

6 Perdew, J. P., Burke, K. & Ernzerhof, M. Generalized gradient approximation made simple. *Phys. Rev. Lett.* **77**, 3865-3868 (1996).

7 Sharafi, A. *et al.* Impact of air exposure and surface chemistry on Li–$Li_7La_3Zr_2O_{12}$ interfacial resistance. *J. Mater. Chem. A* **5**, 13475-13487 (2017).

8 Wagman, D. D., Evans, W. H., Parker, V. B., Schumm, R. H. & Halow, I. The NBS tables of chemical thermodynamic properties. Selected values for inorganic and C_1 and C_2 organic substances in SI units. (National Standard Reference Data System, 1982).

9 Chase Jr, M. *et al.* JANAF thermochemical tables, 1982 supplement. *J. Phys. Chem. Ref. Data* **11**, 695-940 (1982).

10 Kim, J.-W. & Lee, H.-G. Thermal and carbothermic decomposition of Na_2CO_3 and Li_2CO_3. *Metall. Mater. Trans. B* **32**, 17-24 (2001).
